# Influence of Regular Breathing Exercises on Blood Pressure: A Cross-Sectional Study on Young Male Indian Population

**DOI:** 10.7759/cureus.87641

**Published:** 2025-07-09

**Authors:** Sariya Nazim, Amrit Podder, Ashwani Sharma, Jayballabh Kumar

**Affiliations:** 1 Physiology, Teerthanker Mahaveer Medical College and Research Centre, Moradabad, IND

**Keywords:** ageing, blood pressure, body mass index, breathing exercise, hypertension

## Abstract

Introduction

Non-communicable diseases are the major contributors to deaths in our country, while deaths due to cardiovascular diseases (CVDs) play a major role in this. Hypertension (HTN) is associated with almost all cardiovascular morbidities and mortalities. Various studies that were done focusing on middle-aged and older individuals have already proven the beneficial effect of breathing exercises in normalizing blood pressure (BP), but similar studies focusing on young individuals are very few. Our study aimed to establish a relationship between regular breathing exercises and BP phenotypes in young individuals.

Materials and methods

Our cross-sectional study was performed with age and gender-matched young male individuals, where we considered group 1 as the participants who did not do any form of breathing exercises and group 2 as the participants who did regular breathing exercises. The comprehensive convenience sampling method was followed in the study where we measured the body mass index (BMI), BP phenotypes (systolic blood pressure (SBP), diastolic blood pressure (DBP), pulse pressure (PP), mean arterial pressure (MAP), and pulse rate (PR)) of all the individuals after obtaining informed voluntary written consent post-obtaining approval from the institutional committees. We continued including participants in our study till at least one group reached a total of 137 participants.

Results

We found significantly (p < 0.05) higher values of BMI and BP phenotypes in group 1 as compared to group 2. We also found a positive correlation between BMI and BP phenotypes.

Conclusion

Our study concludes that there is an association between regular breathing exercises and lowering of BP in young individuals.

## Introduction

According to data from the World Health Organization, cardiovascular diseases (CVDs) are responsible for 27% of India's non-communicable illnesses (NCDs), which account for roughly 63% of all fatalities. Approximately one-fourth of all CVD cases occur in young people [1]. Over the last 60 years, the prevalence of CVD in India has risen from 2% to 25% among urban residents and from 2% to 15% among rural residents [2]. By 2025, the Indian Hypertension Control Initiative (IHCI) 2020 targets to lower India's hypertension (HTN) patient prevalence [3]. Pranayama is one of the practices that may control blood pressure (BP) by altering hepatic, renal, and cardiac blood flow [4]. By immediately decreasing BP and lowering stress levels, 2:1 breathing may benefit people with essential HTN [5]. Additionally, slow and rapid pranayama may lower diastolic BP, respiratory rate, and heart rate (HR) [6,7]. Cardiovascular functioning is altered by the autonomic regulation of yoga via breathing patterns, which sets off a variety of cerebral and autonomic processes [8]. Pranayama exercises help lower BP in hypertensive individuals and speed up metabolism in overweight people [9,10]. Vascular abnormalities change peripheral resistance and increase vascular stiffness, which are important factors in the development of HTN [11]. Regular breathing exercises significantly help elderly people's BP return to normal, according to recent research [12]. However, there is little data on how regular breathing exercises affect CVD in young people; the majority of research on this topic has been done on middle-aged or older people. The purpose of this research is to contribute to society and IHCI 2020 by establishing a connection between frequent regular breathing exercises and BP in young individuals, so that an early prevention can be achieved, where there is a rising prevalence of HTN-related diseases in this demographic.

## Materials and methods

Study design

Our cross-sectional study which followed the Strengthening the Reporting of Observational Studies in Epidemiology (STROBE) guidelines was conducted from June 2024 to March 2025, at “The Yoga Lab” of the Department of Physiology of Teerthanker Mahaveer Medical College and Research Centre, Teerthanker Mahaveer University, Moradabad, Uttar Pradesh, India, after approval from the College Research Committee (CRC) and Institutional Ethics Committee (IEC) (ref no: TMU/IEC/2024-2025/PG/89, dated June 10, 2024).

Method of collection of data

The participants were chosen using a comprehensive convenience sampling method after obtaining voluntary written informed consent. Our present study's participants were split into two groups, in which group 1 included those who were not engaged in any form of breathing exercises, and group 2 included those who had been engaged in regular breathing exercises for at least three months. Regular breathing exercise was considered for those who are performing any form of breathing exercise for at least 10 minutes, for at least five days a week. We examined 311 male participants for the body mass index (BMI), HR, and BP phenotypes (systolic blood pressure (SBP); diastolic blood pressure (DBP); pulse pressure (PP); mean arterial pressure (MAP)). Male individuals [13,14] in the age group of 18 to 25 years were included as participants in our current study. Any participants with clinical presentations related to cardiovascular, respiratory, renal, and endocrine disorders were excluded from the current study [15]. We also excluded individuals taking any medications that can affect vascular physiology [15], individuals with vascular diseases or with any other chronic diseases [9], and individuals with a history of smoking, alcohol abuse, and tobacco chewing [16]. A digital weighing machine was used for measurement of weight in kg, a stadiometer was used for measurement of height in cm, and a digital sphygmomanometer was used for measurement of BP phenotypes [17] The SBP (mmHg) and DBP (mmHg) were measured in supine position in the morning between 9 AM and 10 AM after 10 minutes of rest to each participants, where three readings were taken with a minimum time interval of 10 minutes each and the average value were considered. The PP and MAP were calculated from the considered value of the SBP and DBP and expressed as mmHg. Quetelet’s index has been employed to compute BMI [18].

Sample size calculation

The sample size was calculated using the following formula [10]:

n = {(σ_1_^2^ +σ_2_^2^) [z_1-α/2_+ z_1-β_]} ÷ (x̄_1_ - x̄_2_)

Where n, samples in each group; x̄_1_, mean of the variable in group 1 (116.78); x̄_2_, mean of variable in group 2 (115.28); σ_1_, standard deviation (SD) of the variable in group 1 (4.59); σ_2_, SD of the variable in group 2 (4.28); z_1-β_ = 0.84 at 80% power of the study; z_1-α/2_ = 1.96 at 95% confidence interval.

Therefore, n = 137.

Thus, total minimum sample = 2 × 137 = 274.

Total sample size of the study

The total analyzed sample size of the study came out as 311, as we continued recruitment of study participants until at least one study group reached the minimum sample of 137 participants. We reached the minimum sample size in each group after the total sample size reached 301, but we considered adding 10 more participants to the total sample size pool to fulfill the secondary objective of the study, which was to measure the percentage of young males practicing regular breathing exercises.

Statistical analysis

The data obtained from the study participants of the current research were analyzed using SPSS software (IBM SPSS Statistics for Windows, IBM Corp., Version 27, Armonk, NY). The data of our study were presented in descriptive and inferential statistics. Mean, SD, frequency, and percentages were evaluated in the descriptive statistics. In inferential statistics, we applied a type 2, two-tailed Student t-test for analysis of comparative variables, where p < 0.05 was considered statistically significant. Pearson’s correlation, with a p-value of less than 0.05, was conducted for the correlation between BMI and the MAP.

## Results

The comparison between all the assessed variables in our study groups is depicted in Table 1. All the participants (n = 311) examined were age-matched. We have observed that the individuals who are performing regular breathing exercises (n = 143) share significantly (p < 0.0001) lower mean values of BP than the non-performing individuals (n = 168). We also found significantly (p = 0.007) higher mean values of BMI in individuals who are not performing regular breathing exercises than in those who are performing regular breathing exercises. This signifies that breathing exercises have a beneficial role in maintaining the BMI of an individual. We also found that 46% of young male individuals are performing regular breathing exercises among the study population. We also found a positive correlation (r = 0.21, p < 0.05) between BMI and MAP among the young male individuals using Pearson's correlation. We did not find any significant difference between the study groups when compared for the HR (p > 0.05).

**Table 1:** Comparison of all assessed variables among groups Group 1: non-performing individuals; group 2: performing individuals; 95% confidence interval of the difference was considered; p-value < 0.05 is considered statistically significant. BMI: body mass index; DBP: diastolic blood pressure; MAP: mean arterial pressure; PP: pulse pressure; SBP: systolic blood pressure

Variables	Group 1 (n = 168)	Group 2 (n = 143)	t-test value	p-value
Age (years)	20.41 ± 1.46	20.49 ± 1.58	-0.318	0.751
SBP (mmHg)	125.97 ± 7.97	114.41 ± 4.18	15.626	<0.001
DBP (mmHg)	76.67 ± 6.89	73.31 ± 4.63	4.954	<0.001
PP (mmHg)	49.31 ± 9.46	41.11 ± 5.78	9.036	<0.001
MAP (mmHg)	93.01 ± 5.73	87.01 ± 3.56	11.018	<0.001
BMI (kg/m^2^)	23.48 ± 4.51	22.12 ± 4.22	2.709	0.007

## Discussion

Our findings show that frequent breathing exercises have a major positive impact on keeping BP and BMI within normal limits. We found that regular breathing exercises have a negative association with lowering the BP, but the mean values of BP of both groups were found to be not in a hypertensive range. This could be the result of several processes that help lessen the negative effects of free radical accumulation, some of which are discussed below.

Role of endothelial dysfunction modulation and inflammation

Maintaining appropriate, optimum endothelial activity protects a physiological vascular architecture. Nitric oxide (NO) degradation occurs when reactive oxygen species (ROS) are produced in excess, which promotes the pathogenic vicious cycle required for vascular endothelial layer dysfunction. As endothelial nitric oxide synthase (eNOS) is stimulated, there is enough NO production, which aids in the preservation of normal vascular architecture. Additionally, this inhibits the pro-inflammatory activity by suppressing the nuclear factor kappa-light-chain-enhancer of activated B cells (NF-kB) signaling pathway [19].

Role of vascular stiffness modulation

The stiffening of the vasculature modifies their architecture, which in turn influences the endothelial cellular biology, resulting in the formation of reduced or lessened elasticity of the vessels. The production of ROS is accelerated by this mechanism, which also raises vascular stress and BP [20]. Breathing exercises assist in maintaining appropriate vascular stress parameters. Consequently, it interrupts the vicious cycle of vascular stiffness development, which maintains BP within a normal range [21-23].

Role of weight management and reduction of obesity

An important risk factor for insulin resistance is obesity, especially abdominal obesity. By lowering stress, boosting metabolism, and promoting mindful eating, breathing techniques may aid with weight management. Eating mindfully improves digestion, reduces hunger, and encourages healthy weight reduction. Reducing excess body fat may greatly increase insulin sensitivity [24]. Frequent breathing exercises help control hunger hormones associated with fullness and appetite, such as leptin and ghrelin. Improving insulin sensitivity aids in weight control, hunger regulation, and preventing overeating. All things considered, consistent breathing techniques may greatly enhance both general health and weight control [25-27].

Comparison with previous studies in a similar direction

According to 2004 research by Pal et al., regular breathing exercises for three months increased vagal activity, which significantly lowered basal HR [28].

In 2011, Sharma et al. came to the same conclusion, demonstrating that breathing exercises may lower HR and BP by increasing vagal activity via a considerable decrease in sympathetic activity and baroreflex system sensitivity [7].

Regular breathing exercises lower diastolic and systolic BP by lowering total peripheral resistance, according to 2009 research by Pramanik et al. and Khan et al. [29,30].

In 2018, Sharma et al. highlighted the way the cardiovascular system's optimum autonomic activity helps maintain BP within a normal range [2].

Considering we observed a similar pattern in our study, our research confirms all of the previously mentioned results in the youth population as well.

Limitations and strengths of the present study

We consider a cross-sectional study design, exclusion of female participants, and self-reporting of regular breathing exercises as some of the limitations of the present study. Convenience sampling introduces selection bias, as participants were recruited from a single center (“The Yoga Lab”), potentially skewing toward health-conscious individuals. This is also to be considered one of the limitations of the study. A young age study population is considered one of the strengths of our study, as there is a rising prevalence of CVDs in this demographic, and early detection and prevention may be possible by practicing regular breathing exercises [30].

## Conclusions

The present study was conducted among 311 young male participants who belonged to the 18-25 years of age range to assess the impact of regular breathing exercises on BP. Our results indicate that regular breathing exercises are associated with lowering the BP of young individuals. Hence, practicing regular breathing exercises for improvement in the adversities related to high BP may be advised in young individuals, although further studies are needed in this direction to observe any influence of breathing exercises on BP.

## References

[1] Kumari R, Rai N, Sharma HB, Kailashiya J (2020). Impact of American Heart Association’s new hypertension guidelines (2017) on disease burden and association with obesity indices. Indian J Physiol Pharmacol.

[2] Sharma A, Misra R, Singh K (2018). Comparison of cardiac autonomic activity between offsprings of normotensive parents and hypertensive parents. Int J Intg Med Sci.

[3] Kaur P, Kunwar A, Sharma M, Durgad K, Gupta S, Bhargava B (2023). The India hypertension control initiative-early outcomes in 26 districts across five states of India, 2018-2020. J Hum Hypertens.

[4] Nivethitha L, Mooventhan A, Manjunath NK (2016). Effects of various prāṇāyāma on cardiovascular and autonomic variables. Anc Sci Life.

[5] Adhana R, Gupta R, Dvivedii J, Ahmad S (2013). The influence of the 2:1 yogic breathing technique on essential hypertension. Indian J Physiol Pharmacol.

[6] Khodnapur JP, Khodnapur GP, Basavaraddi IV (2024). Yoga improves vascular stiffness in COVID-19 survivors of Vijayapur, Karnataka, India. Biomed Pharmacol J.

[7] Sharma VK, Trakroo M, Subramaniam V, Rajajeyakumar M, Bhavanani AB, Sahai A (2013). Effect of fast and slow pranayama on perceived stress and cardiovascular parameters in young health-care students. Int J Yoga.

[8] Ankad RB, Herur A, Patil S, Shashikala GV, Chinagudi S (2011). Effect of short-term pranayama and meditation on cardiovascular functions in healthy individuals. Heart Views.

[9] Jayawardena R, Ranasinghe P, Ranawaka H, Gamage N, Dissanayake D, Misra A (2020). Exploring the therapeutic benefits of “Pranayama” (yogic breathing): a systematic review. Int J Yoga.

[10] Upadhyay Dhungel K, Malhotra V, Sarkar D, Prajapati R (2008). Effect of alternate nostril breathing exercise on cardiorespiratory functions. Nepal Med Coll J.

[11] Naik A, Biswas DA, Patel S (2012). Effect of left nostril breathing in hypertensives. J Indian Acad Clin Med.

[12] Gopal A, Mondal S, Gandhi A, Arora S, Bhattacharjee J (2011). Effect of integrated yoga practices on immune responses in examination stress - a preliminary study. Int J Yoga.

[13] Kwissa M, Krauze T, Mitkowska-Redman A, Banaszewska B, Spaczynski RZ, Wykretowicz A, Guzik P (2022). Cardiovascular function in different phases of the menstrual cycle in healthy women of reproductive age. J Clin Med.

[14] Pal GK, Pal P, Nanda N (2019). Comprehensive Textbook of Practical Physiology. Comprehensive Textbook of Practical Physiology. 4th Ed. Jaypee brothers, Medical Physiology Private Limited, New Pal GK, Pal P. Comprehensive Textbook of Practical Physiology. 4th Ed. Jaypee brothers, Medical Physiology.

[15] Herawati I, Mat Ludin AF, M M, Ishak I, Farah NM (2023). Breathing exercise for hypertensive patients: a scoping review. Front Physiol.

[16] Dandekar PD (2013). Impact of short-term training of anulom vilom pranayam on blood pressure and pulse rate in healthy volunteers. Int J Res Ayurveda Pharm.

[17] Bargal S, Nalgirkar V, Patil A, Langade D (2022). Evaluation of the effect of left nostril breathing on cardiorespiratory parameters and reaction time in young healthy individuals. Cureus.

[18] Podder A, Patil SM, Kanthe PS (2023). Physical anthropometry influences arterial stiffness in hypertensive patients of North Karnataka. Biomed Pharmacol J.

[19] Kumar J, Sharma A, Dasgupta A (2024). Unraveling the relationship between vitamin D and oxidative stress: a cross-sectional study. Cureus.

[20] Sharma A, Patil SM, Dasgupta A, Podder A, Kumar J, Sindwani P, Karumuri P (2024). Unravelling the intricate relationship between oxidative stress and endothelial dysfunction in hypertension. Cureus.

[21] Podder A, Patil SM, Khodnapur JP, Das KK, Badiger S, Kumar J, Adhana R (2025). The intricate relationship of vitamin-D with arterial stiffness: a case control study on middle-aged Indian population with an insight from the mechanistic pathways providing special reference to endothelial dysfunction and oxygen sensing proteins. J Neonatal Surg.

[22] Patil SM, Khodnapur JP, Das KK, Podder A (2024). The role of serum erythropoietin (EPO) and vascular endothelial growth factor (VEGF) in pulse wave velocity (PWV) among hypertensive patients: a cross-sectional study. Cureus.

[23] Ghazvineh D, Daneshvar M, Basirat V, Daneshzad E (2022). The effect of yoga on the lipid profile: a systematic review and meta-analysis of randomized clinical trials. Front Nutr.

[24] Unick JL, Dunsiger SI, Bock BC, Sherman SA, Braun TD, Wing RR (2022). A preliminary investigation of yoga as an intervention approach for improving long-term weight loss: a randomized trial. PLoS One.

[25] Swift DL, Johannsen NM, Lavie CJ, Earnest CP, Church TS (2014). The role of exercise and physical activity in weight loss and maintenance. Prog Cardiovasc Dis.

[26] Podder A, Sharma A, Dasgupta A, Khodnapur JP, Patil SM, Kumar J, Nazim S (2025). Vascular ageing influences arterial stiffness: a cross-sectional study of different age groups with special reference to blood pressure and obesity. J Neonatal Surg.

[27] Chaitanya S, Datta A, Bhandari B, Sharma VK (2022). Effect of resonance breathing on heart rate variability and cognitive functions in young adults: a randomised controlled study. Cureus.

[28] Pal GK, Velkumary S, Madanmohan Madanmohan (2004). Effect of short-term practice of breathing exercises on autonomic functions in normal human volunteers. Indian J Med Res.

[29] Pramanik T, Sharma HO, Mishra S, Mishra A, Prajapati R, Singh S (2009). Immediate effect of slow pace bhastrika pranayama on blood pressure and heart rate. J Altern Complement Med.

[30] Khan IA, Agarwal PK, Paliwal NW (2025). Benefits, myths, and acceptability of yoga. Indian Pract.

